# FANTASIA suite: a reproducible and configurable framework for embedding-based functional annotation of proteins

**DOI:** 10.1093/nargab/lqag106

**Published:** 2026-09-11

**Authors:** Francisco M Pérez-Canales, Àlex Domínguez-Rodríguez, Belén Carbonetto, Rosa Fernández, Ildefonso Cases, Ana M Rojas

**Affiliations:** Evolutionary Dynamics Department, Andalusian Center for Developmental Biology (CSIC-UPO), Seville 41089, Spain; Evolutionary Dynamics Department, Andalusian Center for Developmental Biology (CSIC-UPO), Seville 41089, Spain; Metazoa Phylogenomics & Genome Evolution Lab, Institute of Evolutionary Biology (CSIC-UPF), Barcelona 08003, Spain; Metazoa Phylogenomics & Genome Evolution Lab, Institute of Evolutionary Biology (CSIC-UPF), Barcelona 08003, Spain; Evolutionary Dynamics Department, Andalusian Center for Developmental Biology (CSIC-UPO), Seville 41089, Spain; Evolutionary Dynamics Department, Andalusian Center for Developmental Biology (CSIC-UPO), Seville 41089, Spain

## Abstract

Embedding-based annotation transfer is increasingly used for protein function inference due to protein language models capture sequence, structural, and functional signals that may extend beyond conventional pairwise similarity. However, systematic application of these approaches requires control over model choice, reference composition, lookup parameters, evidence traceability, and output formats. We developed the FANTASIA suite, a configurable framework for embedding-based functional annotation of proteins. The suite combines a database-backed implementation for reproducible and extensible analyses with a portable flat-file implementation for rapid local annotation and pipeline integration. Using non-model and model-organism proteomes, we show that larger neighbourhood sizes remain practical for proteome-scale analyses and that taxonomy and sequence-identity filtering support leakage-aware benchmarking. We also compare the supported models with baseline methods through external CAFA5 evaluation and provide practical guidance based on empirical evidence variables. FANTASIA provides a controlled, scalable, and reproducible framework for extending functional annotation across the rapidly expanding diversity of sequenced organisms.

## Graphical Abstract

**Graphical Abstract:**
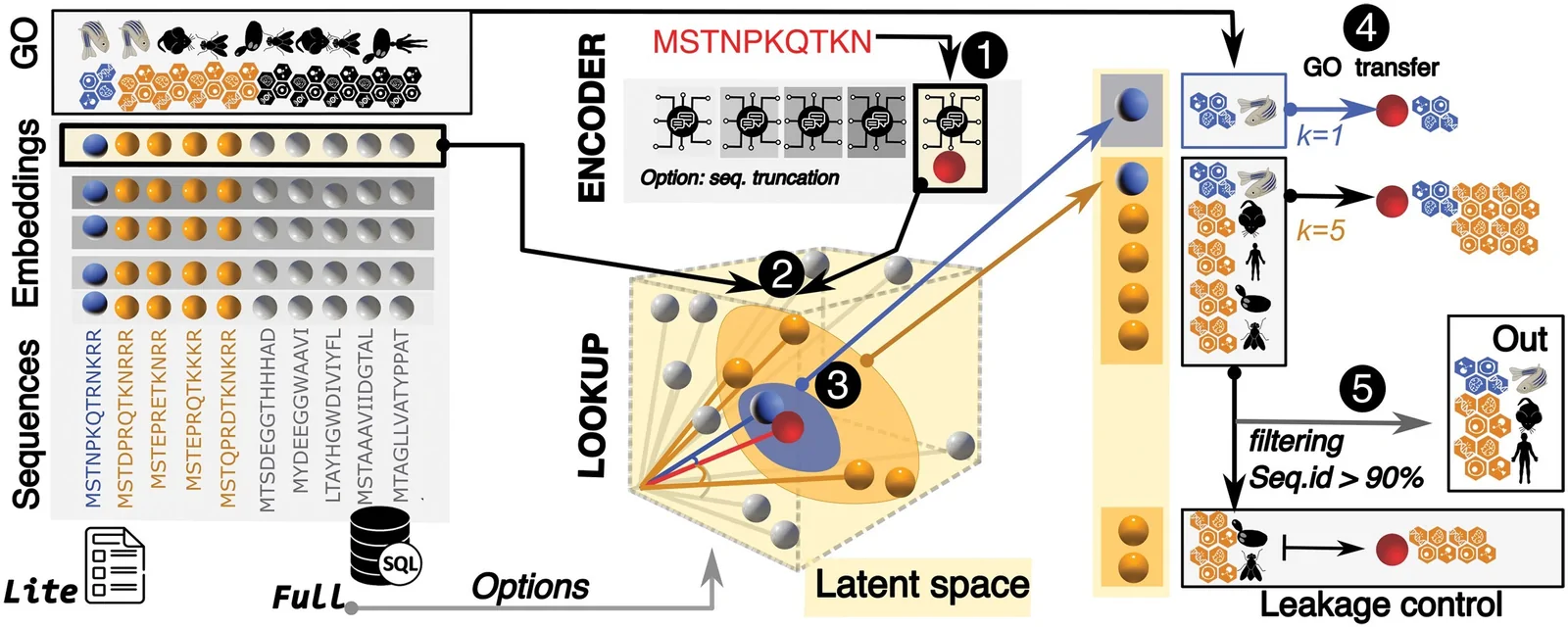

## Introduction

Protein language models (pLMs) transform protein sequences into numerical representations that can be used for prediction, comparison, and annotation [1]. Similarity in embedding space has been exploited to transfer functional annotations to proteins that are difficult to annotate using conventional homology-based methods [2], detect remote homology [3], and recover functional signals in enrichment analyses from transcriptomics data [4]. These observations support the large-scale annotation of genes that are poorly annotated by traditional methods across diverse animal proteomes, enabling downstream biological hypotheses when homology- and pLM-based annotations are compared [5, 6].

Despite this promise, the practical use of embedding-based annotation transfer remains limited by its low reproducibility and configurability. Earlier implementations did not provide systematic control over reference composition, evidence codes, Gene Ontology Annotation (GOA) versions, taxonomy filtering, neighbourhood size, embedding model, layer choice, or output traceability. These limitations are especially important for benchmarking, where reference leakage from identical or closely related proteins can inflate the apparent performance, and for exploratory analyses, where comparisons across models or layers require identical logic execution.

FANTASIA is developed to overcome these limitations. It is not intended to provide a calibrated function-prediction score, but rather a controlled framework for expert interpretation of embedding-based annotation transfer. It enables users to generate or reuse protein embeddings, compare query proteins with an experimentally annotated reference space, transfer Gene Ontology (GO) terms from nearest neighbours, and retain the metadata required to audit each annotation. The suite comprises two complementary implementations: FANTASIA, a database-backed workflow for reproducible annotation, benchmarking, and model comparison; and FANTASIA-Lite, a portable flat-file workflow for rapid local use and pipeline integration. Their technical differences are summarized in Supplementary Table ST1.

## Materials and methods

### System design principles

The FANTASIA suite was designed to support controlled, traceable, and reproducible functional annotation transfer from pLM embeddings.

It is designed around seven principles: modularity, traceability, reproducibility, configurability, auditability, comparative usability, and deployment flexibility. Embedding generation, lookup, post-processing, and orchestration are separated into independent steps. Each run is organized in a self-contained experiment directory containing configuration files, logs, intermediate files, and outputs. The runtime behavior is governed by explicit configuration files, allowing users to swap the pLM, embedding layer, distance metric, lookup threshold, neighbourhood size, taxonomy filters, and post-processinging options without modifying the source code. Therefore, the same workflow can be applied across models, layers, and reference settings while preserving comparability between runs.

**Figure 1.**
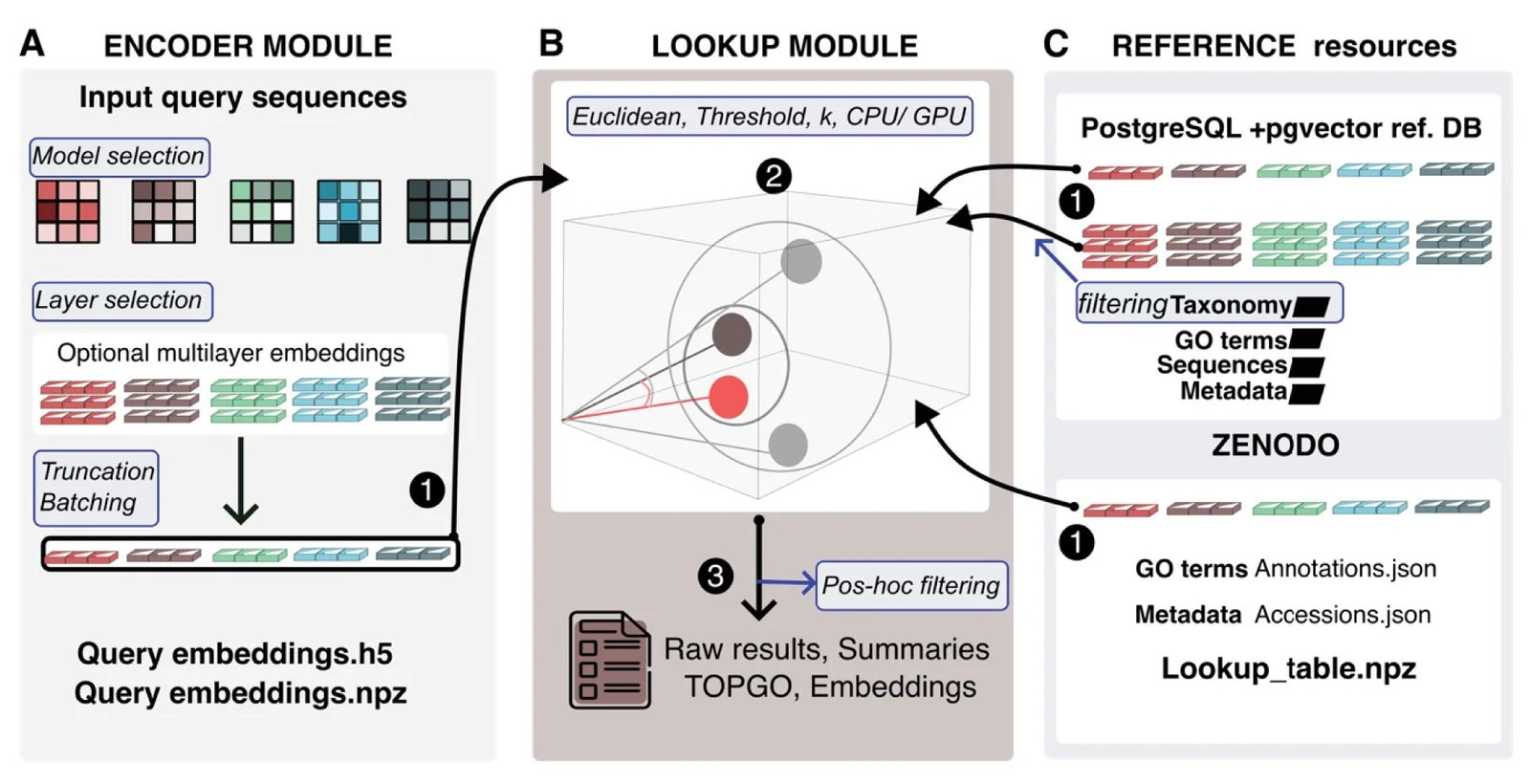
FANTASIA suite workflow. (**A**) ENCODER Module. Input protein sequences are converted into query embeddings using the supported pLMs. Depending on the selected implementation and model, embeddings are stored in HDF5 or NPZ format, and selected models support extraction from multiple network layers. (**B**) Lookup module. Query embeddings are compared with the reference embedding space using a configurable distance metric and neighbourhood size. Optional filters (i.e. taxonomy exclusion) can be applied to the retrieved neighbours, and the workflow produces raw results, annotation summaries, transferred embeddings, and files for downstream enrichment analysis. (**C**) Reference resources. The lookup module accesses reference embeddings together with sequence, taxonomy, accession, and GO metadata. These resources are distributed through Zenodo in either database-backed or flat-file formats. Detailed differences between the two implementations are provided in Supplementary Table ST1. Blue boxes indicate optional configuration.

*FANTASIA v4.1.1* implements a two-stage workflow comprising an ENCODER module and a LOOKUP module (Fig. 1A and B). In the first stage, input protein sequences are embedded using one or more supported pLMs. Query embeddings are stored in HDF5 format and indexed by accession, model, and layer. Embedding generation is independent of annotation transfer, allowing the same embedding archive to be reused for lookup-only analyses, benchmarking, layer comparisons, and exploration of embedding space. An embedding-only mode is also available. Maximum query-sequence length is an explicit, configurable parameter that enables sequence truncation of long proteins; however, truncation can substantially alter neighbour selection and GO transfer for proteins exceeding the cap (Supplementary File 1 and Supplementary Table ST2). Reference-protein embeddings remain full length.

In the second stage, the LOOKUP module (Fig. 1B) compares query embeddings with a reference embedding space and transfers GO annotations from experimentally annotated reference proteins (Fig. 1C). Reference data are distributed as PostgreSQL database dumps with pgvector support and associated biological metadata. PostgreSQL provides structured storage, metadata integration, and controlled retrieval of model- and layer-specific vectors. Large-scale nearest-neighbour calculations are then performed in application memory using batched CPU or GPU operations. This hybrid design combines manageable relational resources with efficient in-memory similarity search.

The full implementation supports explicit control of the lookup universe through taxonomy filters, distance thresholds, neighbourhood size *k*, optional MMseqs2-based redundancy [7] filtering, and optional sequence-aware post-processing. Pairwise sequence alignment between query and donor proteins can be computed using Parasail [8] to provide global and local identity and similarity metrics. These metrics can be used to inspect or filter transferred annotations, particularly in benchmark settings where identical or near-identical reference proteins should be excluded from analysis.

*FANTASIA-Lite V1*. It implements the same conceptual annotation transfer workflow in a standalone, local form. It removes the requirement for PostgreSQL, pgvector, RabbitMQ, and service-backed orchestration. Instead, it uses a static flat-file reference bundle containing pre-computed reference embeddings, GO annotations, and accession metadata. This design simplifies installation and execution while retaining embedding-based nearest-neighbour GO transfer from experimentally annotated proteins. FANTASIA-Lite supports automatic CPU/GPU detection, embedding reuse, optional raw neighbour-level output, optional topGO export, and embedding-only workflows. It is less extensible than the full database-backed platform but is well-suited for rapid local annotation and integration into external pipelines, including BRAKER4 (https://GitHub.com/Gaius-Augustus/BRAKER4). The main aspects of each bundle are listed in Supplementary Table ST1. The reference embeddings were generated using an NVIDIA GeForce RTX 3090 Ti (24 GB VRAM).

### Supported models and reference resources

#### Supported models

FANTASIA currently supports five pLMs: ProtT5, ESM2, ProstT5, Ankh3-Large, and ESMC-600M [9–13]. These models differ in architecture, training data, and representation strategy. ESM2 and ESMC-600M are transformer-based sequence models from the ESM family; ProtT5 and Ankh3-Large use encoder representations derived from T5-style architectures trained on protein sequences; and ProstT5 links protein sequences with structural-alphabet representations, thereby incorporating structural context. These complementary models have different computational profiles and allow annotation behavior to be compared within a controlled workflow (Supplementary File 1). Cosine distance is the default metric because vector magnitudes may differ across models. Euclidean distance is also available but is more sensitive to embedding scale and should be used with appropriate normalization.

#### Reference resources

The reference resources were generated from UniProt [14] and GOA records [15] using a dedicated protein information system workflow (https://GitHub.com/CBBIO/protein-information-system). The current reference comprises 127 546 UniProt protein records, corresponding to 124 397 unique sequences and 627 932 experimental GO annotations. Only experimental or manually supported evidence codes were retained (EXP, IDA, IPI, IMP, IGI, IEP, TAS, and IC). Two reference releases are available: a compact single-layer release containing the final representation layer of each supported model and a larger multi-layer release containing six layers per model for comparison between final and internal representations (see “Data availability” section). Missing embeddings, detailed dataset statistics, model-specific layer lists, and failed sequence reports are provided with the reference repositories. The reference releases contain full-length reference-protein embeddings.

### Outputs

Both implementations produce annotation tables for downstream inspection and optional enrichment analysis, but at different levels of detail. FANTASIA-Lite produces a compact final annotation table, optional raw neighbour-level results, failed-sequence reports, reusable query embeddings, run metadata, and optional topGO-compatible files [6]. FANTASIA v4.1.1 produces more detailed per-run output, including raw query-to-reference-to-GO transfer tables, summary files aggregating support across retrieved neighbours, optional alignment-derived sequence metrics, query embedding archives, and topGO-compatible exports [6]. The summary output (Supplementary Table ST3) is intended for prioritization and inspection. In *k* > 1 runs, support values indicate the fraction of retrieved neighbours contributing a given GO term; they should not be interpreted as calibrated probabilities or universal confidence estimates. Further details are provided in Supplementary File 1.

## Results

### Annotation mode, CPU versus GPU

To illustrate the annotation of a non-model proteome, we used the proteome of *Paratomella rubra* (a non-model invertebrate animal from the phylum Acoela, as an example of a deeply divergent and functionally under-annotated lineage lacking extensive curated genomic resources) [16], which is not represented in the reference database. ProtT5 [9, 17] was selected as the baseline model using cosine distance and *k* = 1. No taxonomy filter or redundancy filter was applied to the data. Query sequences were capped at 2000 aa for testing purposes. The input consisted of 20 223 proteins with a mean length of 392.25 amino acids and a maximum length of 8215 amino acids (only 211 out of 20 223 proteins were longer than 2000 aa). Query embeddings were generated once and then used to compare CPU and GPU lookup performance. This comparison remains internally controlled because both lookup modes reused the same query embeddings.

The CPU/GPU benchmark evaluated only the device used for the lookup distance computation while keeping the precomputed query embeddings constant. On the CPU, the cumulative distance computation required 1835.89 s, with a complete lookup wall time of 1933.08 s, whereas on the GPU, the cumulative distance computation was reduced to 17.05 s, and the total lookup wall time was 126.95 s. Thus, the GPU execution accelerated the vector-distance calculation by ~108-fold and the complete lookup stage by ~15-fold. The difference between the distance computation and full-stage speedup reflects the overhead from data loading, batching, result writing, and post-processinging. These results show that CPU-based lookup is feasible but becomes a bottleneck for large annotation jobs, whereas GPU lookup substantially improves the practical runtime.

The benchmark was performed on a workstation equipped with an NVIDIA GeForce RTX 3090 Ti GPU with 24 GB of VRAM. CUDA was available in the runtime environment, and the PyTorch build used for the benchmark was 2.11.0 + cu130.

### Benchmark/leakage-control mode

To illustrate the leakage-aware benchmarking on speed performance, we used the *Mus musculus* proteome, a model organism that is expected to be heavily represented in the reference database. From the UniProtKB (UniprotKB proteomes on 18-04-2026, UP000000589) proteome, we selected 21 852 proteins, one for each gene. Query embeddings were uncapped. We additionally tested whether embedding truncation had an effect on the lookup process by truncating sequences >2000 aa, and found that the effect on GO transfer and neighbour assignment is substantial (see Supplementary Table ST2) for longer proteins.

#### Baseline run

A baseline ProtT5 run with a cosine distance and *k* = 1 was used to mimic a simple annotation mode in *Mus musculus*. This run required 33 min 29 s in total, including 26 min 45 s for embedding generation and 6 min 45 s for lookup. Of the 21 852 input proteins, 21 830 were successfully embedded and 22 failed during uncapped embedding. Adding a taxonomy filter to exclude mouse reference sequences from the reference database increased the lookup time to 7 min 27 s, indicating that taxonomy-aware exclusion remains computationally modest at this scale.

#### Neighbourhood expansion

We then reused the same query embeddings (from untruncated sequences) to evaluate larger neighbourhoods in ProtT5. Increasing the lookup from *k* = 1 to *k* = 5 increased the lookup time from 6 min 45 s to 9 min 57 s, corresponding to 0.01855 and 0.02735 s per protein, respectively. This increase was moderate and remained practical for proteome-scale analyses. Across all five supported models, *k* = 5 workflows were completed within ~26–39 min on the test workstation (Supplementary Table ST4). Embedding generation was the dominant runtime component for all models, whereas lookup contributed a smaller but still substantial fraction of the runtime. ESMC-600M was the fastest overall, whereas Ankh3-Large was the slowest, mainly because of its longer embedding time.

#### Post-hoc filtering

For data leakage control, taxonomy filtering at lookup can be combined with post-hoc sequence identity filtering of the retrieved neighbours (Supplementary File 1). In the mouse *k* = 5 benchmark, global identity filtering produced broadly consistent behavior across models (Supplementary Table ST5). At stringent identity cutoffs, a small number of query proteins lost all surviving donor proteins after filtering, reflecting the limited candidate set available within *k* = 5. Larger neighbourhoods may recover acceptable donors for some of these cases. These results support *k* = 5 as a practical compromise for benchmark analyses because it retrieves multiple candidate donors while still allowing the removal of near-self or high-identity reference proteins after a lookup. A description of the information provided in the results is presented in Supplementary Table ST6.

### Cross-model comparison and model external validation

To determine whether the supported models produce similar functional annotations and select the same donor proteins, we calculated Wang semantic similarity [18] and donor-set Jaccard overlap. The models produced highly similar, but not identical, annotations from partially different donor sets (Supplementary Fig. SF1 and Supplementary Table ST7). This contrast was strongest in worm, which is less densely represented in the reference database.

To assess whether FANTASIA performance depends strongly on neighbourhood size, we benchmarked *k* = 1, 3, 5, and 10 for all five models against the three CAFA5 test sets (no knowledge, limited knowledge, and partial knowledge) across all GO namespaces (Supplementary Fig. SF2A–E and Supplementary Table ST8) [19]. Performance was generally insensitive to *k* within this range. The *k* = 3 setting provided a useful overall balance and, in several no-knowledge and limited-knowledge panels, approached the CAFA5 ProtT5 baseline (Supplementary Fig. SF3). All configurations showed a similar decline under the partial-knowledge setting (Supplementary Files 1 and 2).

### General validation and practical interpretation of predictions

To provide practical guidance for interpreting FANTASIA annotations, we evaluated annotation transfer across 12 500 sequence-diverse proteins arranged into five genus-disjoint design panels (see Supplementary File 2 for a comprehensive description, and Supplementary Table ST9).

**Figure 2.**
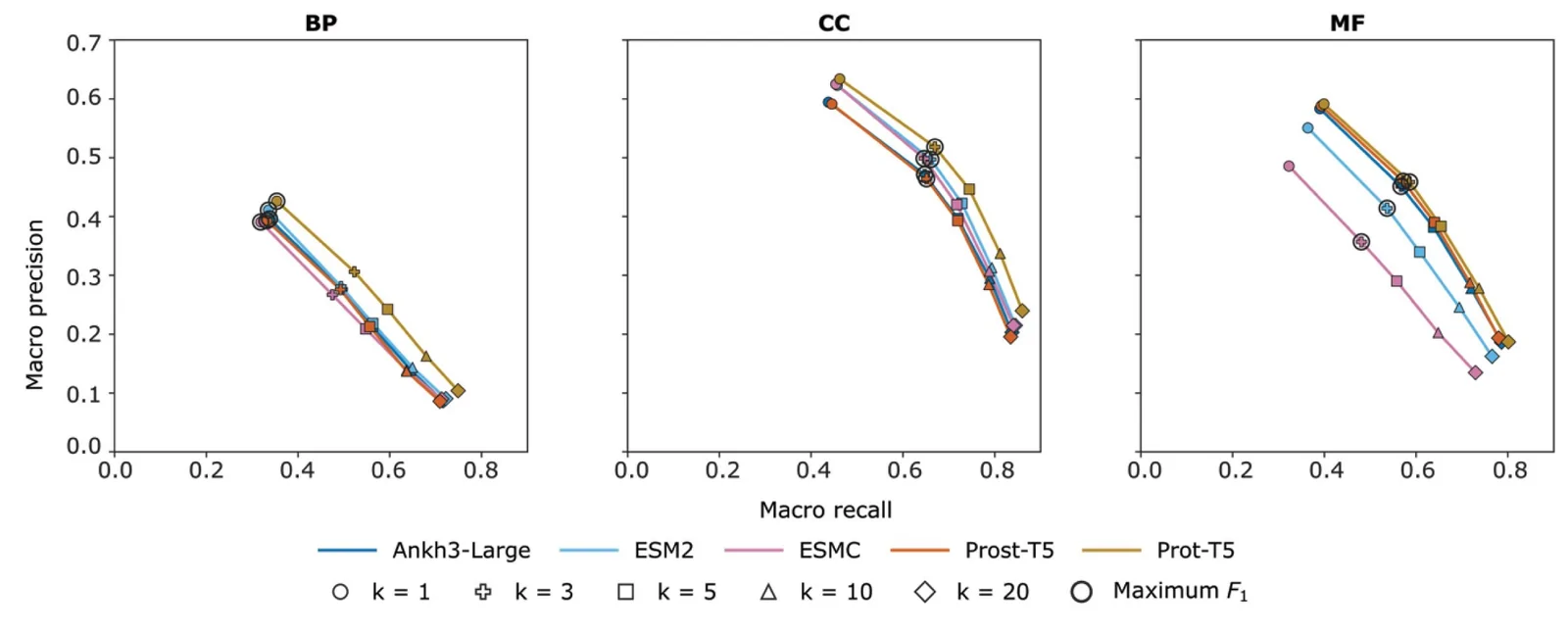
Macro precision-recall trade-offs at *k* = 1, 3, 5, 10, and 20 for five pLMs, stratified by GO. Lines connect *k* values within a model; marker shape identifies *k*. An open charcoal ring marks the maximum F1 among the displayed *k* values for each model-GO aspect curve. Precision and recall are protein-macro averages, giving each protein equal influence within its GO aspect.

**Figure 3.**
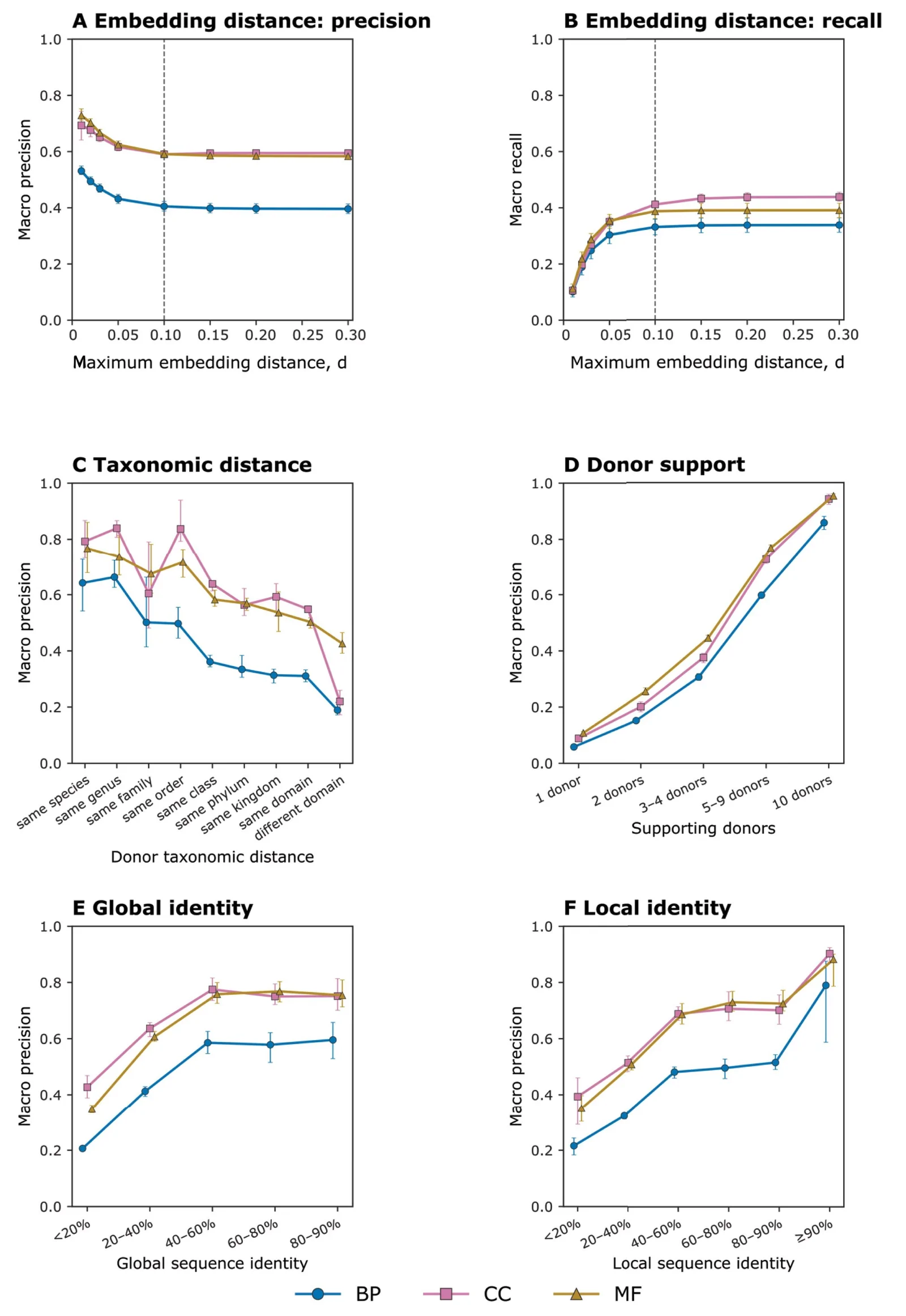
Empirical evidence profiles for Ankh3-Large, stratified by GO. Macro precision (**A**) and recall (**B**) at *k* = 1 under cumulative embedding-distance thresholds; the dashed reference line marks *d* = 0.10. (**C**) Macro precision across exact donor taxonomic-distance categories at *k* = 1. (**D**) Macro precision across disjoint supporting-donor bins at *k* = 10. Macro precision across disjoint global (**E**) and local (**F**) sequence-identity bins at *k* = 1. Colors and marker shapes identify GO aspect; whiskers show 95% whole-panel intervals across up to five genus-disjoint design panels. Ankh3-Large was selected for visualization because it had the smallest equal-panel mean absolute deviation from the cross-model medians across the six displayed metrics (1.40 percentage points); this is a representativeness criterion rather than a model-performance ranking. Full five-model results are shown in Supplementary Figs SF4–SF9. Taxonomic categories are exact bins rather than cumulative thresholds, and no global-identity observations were available in the ≥90% bin (since they were excluded in the construction panel).

The choice of *k* depends on the intended application (Fig. 2 and 3A and B; Supplementary Figs SF4 and SF5). The *k* = 1 setting is the most conservative, whereas *k* = 3 provides a useful balance between precision and coverage, particularly for MF and CC. Values of *k* = 3 or *k* = 5 are preferred for recall-oriented applications such as GO-term enrichment, while larger *k* are better suited for exploratory purposes.

For conservative annotation transfer, we recommend *k* = 1 and prioritizing predictions associated with the shortest embedding distances. For most model-ontology combinations, an embedding distance around 0.10 marked a useful empirical transition, beyond which broader thresholds produced relatively modest gains in recall and little additional precision. ProtT5 followed a different pattern, with precision declining more gradually across the evaluated distance range; therefore, this value should be interpreted as a practical prioritization guide rather than a universal cutoff (see details in Supplementary File 2).

Taxonomic proximity was also informative, particularly across distant-to-intermediate donor relationships (Fig. 3C and Supplementary Fig. SF7). For MF and CC, most of the observed improvement was reached by approximately the order level, although the pattern was not strictly monotonic across all taxonomic categories. For *k* > 1, the number of independent supporting donors showed the clearest empirical association with annotation agreement (Fig. 3D and Supplementary Fig. SF6). Predictions supported by several donors therefore represent particularly informative subsets, although these subsets become progressively smaller as the support requirement increases.

Global sequence identity showed its largest increase in discriminatory value between the lowest-identity categories and the 40%–60% range (Fig. 3E and Supplementary Fig. SF8), with more modest gains at higher identity levels. Local sequence identity retained an additional high-similarity signal, consistent with cases in which conserved motifs or domains support annotation transfer despite lower full-length similarity (Fig. 3F and Supplementary Fig. SF9).

Overall, predictions supported by several complementary features, including short embedding distance, closer taxonomic relationships, higher global or local sequence identity, and repeated donor support, should be prioritized. These variables provide empirical evidence for ranking predictions but should not be interpreted as calibrated probabilities of correctness. Predictions lacking these features may still be biologically relevant and should be treated as lower-priority or unconfirmed annotations.

### Practical recommendation

For conservative annotation transfer, we recommend *k *= 1 and prioritizing predictions with short embedding distances, while treating *d* ≈ 0.10 as an empirical guide rather than a universal cutoff. For broader-coverage applications, including enrichment analyses, *k *= 3 or *k *= 5 may be used, with predictions ranked using complementary donor-support, taxonomic, and sequence-similarity evidence.

## Discussion

FANTASIA extends previous embedding-based GO annotation transfer approaches [2, 17] by enhancing the configurability, reproducibility, and auditability. Rather than providing a single fixed annotation score, it enables a systematic comparison of pLMs, layers, lookup settings, taxonomy filters, and sequence-based post-processing rules.

The two implementations serve different use cases and are not interchangeable. FANTASIA v4.1.1 is intended for controlled research workflows, benchmarking, reference management, and comparative analyses across models and layers. FANTASIA-Lite sacrifices some database-backed flexibility in favor of portability and ease of deployment, making it suitable for rapid annotation and pipeline integration. Together, these implementations provide a practical route for embedding-based GO transfer in both exploratory and assignment settings in the future. Sequence-length handling is optional but requires careful consideration. Although truncation may be necessary for very long sequences, it alters their representations and, as shown here, can affect GO transfer. A full-length reference embedding library remains useful under both capped and uncapped configurations.

Cross-model agreement was higher in mouse, particularly for MF and CC, than in worm, where MF and BP varied more across models. Donor overlap was consistently lower than semantic agreement, indicating that different models often selected different donors while transferring related GO functions. The lower worm concordance may reflect a more heterogeneous reference neighbourhood after self-species exclusion and does not imply lower annotation accuracy.

FANTASIA does not assign scores or probabilities to individual GO-term predictions. Previous analyses have shown that reliability scores [2] derived from embedding distances do not necessarily correlate with the semantic similarity between the predicted and true GO terms [4]. Such scores still recover useful signals at the aggregate level [5], as also observed in our internal evaluation. External validation using CAFA5 datasets and evaluation tools further showed that FANTASIA performs as expected. In addition, our systematic assessment of output features provides a practical framework for prioritizing annotations. Together, these results support FANTASIA as a robust annotation-transfer framework that facilitates prioritization and expert interpretation without implying calibrated probabilistic confidence for individual predictions.

In summary, FANTASIA complements existing function-annotation methods by providing a transparent framework for embedding-based annotation transfer. It is particularly useful when users seek to expand annotation coverage for downstream statistical analyses, compare models, control reference leakage, inspect transferred evidence, or generate topGO-compatible outputs. Its principal contribution is to make embedding-based annotation workflows more reproducible and interpretable, rather than to replace expert curation or calibrated prediction methods.

## Supplementary Material

lqag106_Supplemental_Files

## Data Availability

The benchmark datasets and analyses of this work are available at Zenodo under the concept DOI, https://doi.org/10.5281/zenodo.20305840. The FANTASIA bundles and lookup tables presented in this article are available at GitHub: •FANTASIAv4.1.1:https://github.com/CBBIO/FANTASIA/tree/fantasia-4.1.1 •FANTASIA-Lite.V1.1:https://github.com/CBBIO/FANTASIA-Lite/tree/FANTASIA-Lite-V1.1 The FANTASIA lookup tables presented in this article are available at Zenodo: •FANTASIA v4.1.1 bundle multi-layer:https://zenodo.org/records/17793273 •FANTASIA v4.1.1 bundle last-layer:https://zenodo.org/records/17795871 •FANTASIA-Lite V1 bundle last-layer:https://zenodo.org/records/19742926
