# Supplementary material for: FANTASIA suite: a reproducible and configurable framework for embedding-based functional annotation of proteins
Source: NAR Genom Bioinform. 2026 Sep 11;8(3):lqag106. doi: 10.1093/nargab/lqag106 (PMC13563086; doi:10.1093/nargab/lqag106)
Supplement: Supplementary file 1 [file lqag106_supplemental_files.zip › Supplementary_File_1_correctedAGO2026.docx]

**SUPPLEMENTARY FILE 1**

**FANTASIA suite: a reproducible and configurable framework for embedding-based functional annotation of proteins**

Francisco M. Pérez-Canales^1†^, Àlex Domínguez-Rodríguez^1†,^ Belén Carbonetto^2^, Rosa Fernández^2,^*, Ildefonso Cases^1*^, and Ana M. Rojas^1,^*

^1^ Evolutionary Dynamics Department, Andalusian Center for Developmental Biology (CSIC-UPO), Seville, 41089, Spain

^2^ Metazoa Phylogenomics & Genome Evolution Lab, Institute of Evolutionary Biology (CSIC-UPF), Barcelona, 08003, Spain

* To whom correspondence should be addressed. Email: a.rojas.m@csic.es. Correspondence may also be addressed to Rosa Fernández and Ildefonso Cases.

^†^ equal contribution.

**Summary:**

FANTASIA’s purpose is to support expert interpretation of embedding-based functional annotation by providing a configurable framework for evaluating embedding similarity, sequence similarity, reference selection, and GO transfer. This file describes the supported models, reference resources, configuration options, outputs, cross-model concordance, and external CAFA5 evaluation. The independent internal evaluation of empiric variables and practical interpretation is provided in Supplementary File 2.

FANTASIA availability:

FANTASIA v4.1.1: <https://github.com/CBBIO/FANTASIA/tree/fantasia-4.1.1>

FANTASIA-Lite.V1.1: <https://github.com/CBBIO/FANTASIA-Lite>

**1- Supported models**

The five supported protein language models provide complementary representation strategies and computational trade-offs.

1) ProtT5 (ProtT5-XL-UniRef50) uses the T5 architecture and was trained on UniRef50 with a masked-language-modelling objective. We used the encoder-only model Rostlab/prot_t5_xl_half_uniref50-enc. It has 24 encoder layers, approximately 1.2 billion parameters, and produces 1,024-dimensional embeddings (1).
2) ESM2 (facebook/esm2_t33_650M_UR50D) is a general-purpose transformer baseline for protein representation learning. It has 33 layers, approximately 650 million parameters, and produces 1,280-dimensional embeddings (2).
3) ProstT5 is a bilingual protein language model that links amino-acid sequences with structural-alphabet representations. The encoder has 24 layers, approximately 1.2 billion parameters, and produces 1,024-dimensional embeddings. The model was trained using approximately 17 million proteins with high-quality predicted structures and was accessed as Rostlab/ProstT5 (3).<https://huggingface.co/Rostlab/ProstT5>
4) Ankh3-Large is a transformer-based encoder optimised for protein representation learning. It has 48 encoder layers, approximately 1.15 billion parameters, and produces 1,536-dimensional embeddings. The model was accessed as ElnaggarLab/ankh3-large (4).
5) ESMC-600M (EvolutionaryScale/esmc-600m-2024-12) is a 36-layer transformer trained with a masked-token objective on a large and diverse protein-sequence collection. It has approximately 600 million parameters and produces 1,152-dimensional embeddings (5).

**2-The lookup reference database**

The reference embeddings are shared as a PostgreSQL database dump in Zenodo. There are two versions: one with only the final hidden layer of each protein language model (<https://zenodo.org/records/17795871>) and a larger one with both intermediate and final layers (<https://zenodo.org/records/17793273>). The Zenodo bundle for FANTASIA-Lite static files derived from the smaller dump is also available (<https://doi.org/10.5281/zenodo.19742925>). These lookup tables were generated using PIS v3.1.0 (Protein Information System at CBBIO, <https://GitHub.com/CBBIO/protein-information-system>), an integrated workflow designed for the automated extraction, processing, and management of protein-related data. PIS efficiently retrieves and integrates information from UniProt, PDB, and GOA, facilitating the acquisition of sequences, structures, and relevant annotations. This framework allows the creation of a custom reference dataset using the proposed schema. All reference datasets were extracted from UniProt (6) as of December 2025 and were limited to entries with experimental evidence codes only (EXP, IDA, IPI, IMP, IGI, IEP, TAS, and IC). It comprises 127,546 UniProt accessions (627,932 GO annotations). Details of the dataset are available in the Zenodo repositories. These dumps were restored to a PostgreSQL database with pgvector support, along with the associated biological metadata, enabling the structured storage, curation, and controlled retrieval of the reference space. However, at runtime, FANTASIA does not use PostgreSQL as the primary engine for large-scale nearest-neighbour computations. Instead, the relevant reference vectors are selected from the database by model and layer, and then loaded into the application memory, where batched similarity calculations are performed on either the CPU or GPU. <https://zenodo.org/records/17795871>; <https://zenodo.org/records/17793273>; <https://doi.org/10.5281/zenodo.19742925>.

**3- Configurability and optional processing**

The primary strength of FANTASIA v4.1.1 is its configurability. The parameters available for configuration include:

- the choice of model(s) to be utilized.
- the number of nearest neighbours (k) to investigate.
- a distance threshold for retrieving donor proteins.
- a specific layer from a multi-layer reference dataset.
- the option to filter sequences by taxonomy ID.
- Additionally, a query sequence-aware redundancy filter using MMseqs2
- can optionally cluster input sequences with the reference dataset by calculating distances by excluding all but the representative protein in each cluster.
- A sequence truncation to cap embedding generation.

Although we provide this option, we advise against using the MMseqs2 option to filter out highly similar hits, as it relies on external tools, and we cannot guarantee the removal of very similar sequences owing to the different clustering processes. To assist users in filtering very similar sequences when k>1 is selected, we included a pairwise sequence alignment step between the query and embedding-donor proteins using Parasail (8). This computes sequence similarity and identity using both global and local approaches, enabling additional filtering strategies based on sequence similarity.

Sequence truncation affects GO transfer for proteins exceeding the selected cap. In mouse, setting max_sequence_length: 2000 left the GO sets of all 21,390 shorter proteins unchanged. Among the 462 proteins longer than 2,000 amino acids, 257-349 GO sets changed, depending on the model (Supplementary Table ST2). Uncapped embedding failed for no long proteins with ESMC-600M, one with ESM2 and Ankh3-Large, and 22 with ProtT5 and ProstT5. Among long proteins successfully processed in both conditions, removal of the cap changed at least one of the five selected donors for 63.6-81.6% of proteins and changed the aggregate transferred GO-term set for 55.6-79.3%. These exact-set changes do not necessarily indicate poorer annotations because alternative terms may be biologically related or occupy nearby positions in the GO hierarchy.

**4- Documentation**

**FANTASIA v4.1.1.** The repository README file provides the main operational guide, including supported protein language models, reference datasets, execution modes, Docker-based service setup, troubleshooting, output interpretation, and citation information. The default YAML configuration and model-specific example configurations document the available runtime options, including model selection, layer selection, lookup parameters, taxonomy filtering, redundancy masking, and post-processinging settings. Additional Sphinx documentation covers the methodological background, system architecture, deployment in local and cluster environments, and result interpretation. Benchmark and annotation examples are included as reproducible test workflows, allowing users to run full-pipeline, lookup-only, and embedding-only analyses.

**FANTASIA-Lite V1.1** The documentation explains the required Zenodo lookup bundle, composed of lookup_table.npz, annotations.json, and accessions.json, including also a bundle with all three tables, and describes how these files provide the reference embedding space, GO annotation layer, and accession mapping. It also documents the default fast workflow based on ProtT5 embeddings, cosine lookup, *k* = 1, full precision, and automatic CPU/GPU selection, together with advanced options for larger k, raw neighbour-level outputs, optional topGO(9) file formats export, embedding reuse, GPU-aware tuning, and embedding-only layer export workflows.

**5- Batching settings**

Batching is used to control GPU intensive processes. There are three batching settings:

- workflow management during embedding generation: input sequences are into queue batches of 100 proteins.
- a model-level batch size of 1, within each embedding worker, proteins are passed to the selected protein language one protein embedded at a time. This ensures robust execution across models and variable protein lengths, minimizing memory-related failures.
- Lookup batching, independent of embedding generation, uses batches of 516 query embeddings for batched distance computation.

**6- Data leakage control and post-filtering processes**

Because Mus musculus is extensively represented in the reference resource, unfiltered benchmarking can retrieve self-species or closely related donors and inflate apparent performance. Taxonomy-based exclusion removes specified taxa before neighbour selection, whereas sequence-identity filtering provides an additional, complementary control for highly similar donors that remain after taxonomy filtering.

Explicit global sequence identity filtering of the *k* = 5 lookup outputs yielded highly consistent results across all tested models (Supplementary Table ST5). At a global sequence-identity cutoff of 0.90, approximately 28.3-28.6k distinct reference proteins were excluded across the five models, while 21,273-21,327 distinct reference proteins remained selected as donors. Between 503 and 578 query proteins were left without any surviving donor. Relaxing the cutoff to 0.95 reduced the number of excluded reference proteins to approximately 21.9-22.1k and the number of queries without a surviving donor to 181-203, while 21,635-21,662 distinct reference proteins remained selected. Despite variation in the total number of raw rows, these outcomes were consistent across models, suggesting that post-hoc leakage-control behavior was largely model-independent in this benchmark. Because filtering was applied only to the retrieved *k* = 5 neighbourhood, larger neighbourhoods (i.e., *k* = 10) might recover acceptable donors for some queries that otherwise lose all candidates.

To help users with filtering after *k* >1, we provide a helper script (filter_raw_results_by_identity.py) that computes post-filtering based on sequence identity and retains the best remaining donor with the highest “reliability_index” after filtering. This measure was first defined in the Gopredsim method (10) to produce a scaled version of the distance result (from 0 to 1, with being 1 the closest). Because it is highly intuitive, we retained it. However, it should be noted that on certain occasions, these values can be model-dependent, organism-dependent, and likely skewed.

**7-Outputs**

*FANTASIA-Lite V1.1* is designed for direct annotation and produces a compact set of files producing the following outputs:

- results.csv is the final GO annotation table. Each row corresponds to a retained GO term after consolidation, making this the primary file for biological interpretation.
- raw_results.csv**.** Optional if k = 1, it is produced when *k*>1 (k.<N>.results.csv) preserves the nearest-neighbour output, including hit rank, reference match, distance, reliability index, GO term, namespace, and evidence codes. This file is useful for inspecting neighbour structure or the effect of retaining multiple hits.
- failed_sequences.csv. It reports skipped sequences.
- query_embeddings.npz (NumPy format).
- execution files: in fantasia_config.yaml, run_metadata.yaml, and run.log.
- Optional topGO-compatible files are written under topgo/.

*FANTASIA v4.1.1.* provides a more detailed output structure for benchmarking, model/layer inspection, and downstream evidence integration:

- raw_results.csv. Raw annotations are written as per-accession CSV files under raw_results/{model}/layer_{k}/, where each row represents one GO term transferred from a retrieved reference hit to a query protein. These files include the query accession, GO identifier and description, namespace, embedding distance, reliability index, model and layer, reference-protein metadata, evidence code, and sequence lengths. When sequence-aware output is enabled, they also include global and local alignment-derived metrics such as identity, similarity, alignment score, gap percentage, and aligned length. Sequence alignments are performed with Parasail (8) (which is embedded within the code). A detail explanation of all the information provided in the raw_results.csv is provided in Supplementary Table ST6. A helper script is provided to merge the results.
- Mappings needed to interpret summarized annotations which are provided in the summary.csv file. This file reports GO-term assignments per query accession and lists the supporting reference proteins, while the raw result tables retain the detailed query-to-reference-to-GO relationships from which the summary is derived (see Supplementary Table ST3). When generated, query_index_mapping.csv provides an additional mapping between internal query indices and the original sequence identifiers.
- query_embeddings HDF5 format.
- Traceability and provenance are supported by per-run experiment directories, saved configurations, generated outputs, and runtime logs; however, failed-sequence handling is recorded through execution logs rather than a dedicated default failure table.
- Optional topGO-compatible files under topgo/, either separated by model, layer, and GO category or as ensemble files retaining the best reliability index across models and layers.

**8- Model overlapping**

The five supported protein language models were compared independently within the mouse and worm proteomes. The same uncapped embeddings generated with the full FANTASIA implementation were reused for cosine-distance lookup with *k* = 5. Reference proteins from the query species were excluded before neighbour selection using taxonomy filters: taxon 6239 for *C. elegans* and taxa 10090 (*M. musculus)*, 10091, and 57486 for other mouse species. MF, BP, and CC annotations were analysed separately. For each protein and GO aspect, all ten pairwise model comparisons were evaluated. Comparisons were included only when both models assigned at least one GO term in the corresponding aspect; comparisons containing an empty GO-term set were treated as missing. For every evaluable comparison, BMA Wang semantic similarity and donor-set Jaccard overlap were calculated, and protein-level scores were summarised by their median

$${\overset{̃}{S}}_{A,B,o}=\mathrm{median}_{p\in E_{A,B,o}}\left( S_{p,A,B,o} \right),$$

Where $E_{A,B,o}$ is the set of evaluable proteins for models $A$ and $B$ in ontology $o$ and $S$ denotes either BMA Wang similarity or donor-set Jaccard. Results are shown in Supplementary Figure SF1, and Supplementary Table ST7.

**9- EXTERNAL CAFA-5 evaluation.**

FANTASIA performance was evaluated across GO namespaces and neighbourhood sizes (*k* = 1, 3, 5, and 10) using the three CAFA5 test sets: no knowledge, limited knowledge, and partial knowledge (11) and the reliability score (10) as the score.

The CAFA5 training set was used as the lookup reference. Embeddings and nearest-neighbour rankings were generated with FANTASIA and converted to the FANTASIA-Lite input format for GO transfer. The distance-derived reliability index was supplied as the prediction-ranking score required by the CAFA evaluator; this use does not imply calibrated probabilities.

Evaluation was performed with CAFA-evaluator-PK (<https://github.com/claradepaolis/CAFA-evaluator-PK>, (12)) using the parameters and auxiliary files reported in the CAFA5 study.

Results of the evaluation are shown in Supplementary Figures SF2A-E and SF3. It is **important to note that the CAFA5 baseline is not a fixed configuration**: depending on the panel it corresponds to different embedding distance metrics (L2 or cosine) and different neighbour counts (3-NN in most panels, but 5-NN for MF Fmax/Partial and 2000-NN for BP/CC Smin/Partial), whichever configuration achieved the best reported score, so it should be read as an upper-bound reference point rather than a like-for-like k comparison. The companion data use to generate the figures are in Supplementary Table ST8.

**The independent internal evaluation and practical interpretation** of FANTASIA empirical variables are provided in Supplementary file 2.

**Supplementary Figure SF1**. **Within-proteome agreement among protein-language models**. Pairwise agreement among models for mouse and worm annotations (Full FANTASIA) using uncapped full-length embeddings, cosine distance, and *k* = 5. Reference proteins from the query species were excluded before neighbour selection. **(A)** mouse BMA Wang semantic similarity, **(B)** worm BMA Wang semantic similarity, **(C)** mouse donor-set Jaccard index, and (**D)** worm donor-set Jaccard index. Each ontology is shown separately. Each off-diagonal cell is the median protein-level score across proteins with at least one GO term from both models in the corresponding category. BMA Wang similarity measures semantic agreement between the transferred GO-term sets, whereas donor-set Jaccard measures exact overlap between the category-specific sets of contributing donor protein IDs. Both metrics range from 0 to 1, with larger values indicating greater agreement. Diagonal cells represent model self-comparisons and were fixed to 1; their numerical labels were omitted for clarity. Cell values are displayed to one decimal place, while the colour scale and all calculations use the unrounded values.

**Supplementary Figure SF2A.** **Individual CAFA5 Evaluation of FANTASIA predictions for Ankh3-Large across neighbourhoods.** *k* = 1, 3, 5, 10. The hatched "CAFA5 ProtT5 Baseline" bars show the best-reported ProtT5 embedding-based baseline from the CAFA5 evaluator for each panel, for reference. This baseline is not a fixed configuration: depending on the panel it corresponds to different embedding distance metrics (L2 or cosine) and different neighbour counts (3-NN in most panels, but 5-NN for MF Fmax/Partial and 2000-NN for BP/CC Smin/Partial), whichever configuration achieved the best reported score, so it should be read as an upper-bound reference point rather than a like-for-like k = 3 comparison.

**Supplementary figure SF2B.** **Individual** **CAFA5 Evaluation of FANTASIA predictions for ESM2 across neighbourhoods.** Elements are the same as figure SF2A but for different *k* = 1, 3, 5, 10.

**Supplementary figure SF2C.** **Individual** **CAFA5 Evaluation of FANTASIA predictions for ESMC-600M across neighbourhoods.** Elements are the same as figure SF2A but for different *k* = 1, 3, 5, 10.

**Supplementary figure SF2D.** **Individual** **CAFA5 Evaluation of FANTASIA predictions for ProstT5 across neighbourhoods.** Elements are the same as figure SF2A but for different *k* = 1, 3, 5, 10.

**`**

**Supplementary figure SF2E.** **Individual** **CAFA5 Evaluation of FANTASIA predictions for Prot-T5 across neighbourhoods.** Elements are the same as figure SF2A but for different *k* = 1, 3, 5, 10.

**Supplementary Figure SF3. Summary CAFA5 Evaluation of FANTASIA predictions for all models**. FANTASIA’s performance was evaluated across GO namespaces k = 3 for all models. The hatched "CAFA5 ProtT5 Baseline" bars show the best-reported ProtT5 embedding-based baseline from the CAFA5 evaluator for each panel, for reference. This baseline is not a fixed configuration: depending on the panel it corresponds to different embedding distance metrics (L2 or cosine) and different neighbour counts (3-NN in most panels, but 5-NN for MF Fmax/Partial and 2000-NN for BP/CC Smin/Partial), whichever configuration achieved the best reported score, so it should be read as an upper-bound reference point rather than a like-for-like *k* = 3 comparison.

**REFERENCES FOR SUPPLEMENTARY MATERIAL**

1. Elnaggar,A., Heinzinger,M., Dallago,C., Rehawi,G., Wang,Y., Jones,L., Gibbs,T., Feher,T., Angerer,C., Steinegger,M., *et al.* (2022) ProtTrans: Toward Understanding the Language of Life Through Self-Supervised Learning. *IEEE Transactions on Pattern Analysis and Machine Intelligence*, **44**, 7112–7127.

2. Lin,Z., Akin,H., Rao,R., Hie,B., Zhu,Z., Lu,W., Smetanin,N., Verkuil,R., Kabeli,O., Shmueli,Y., *et al.* (2023) Evolutionary-scale prediction of atomic-level protein structure with a language model. *Science*, **379**, 1123–1130.

3. Heinzinger,M., Weissenow,K., Sanchez,J.G., Henkel,A., Mirdita,M., Steinegger,M. and Rost,B. (2024) Bilingual language model for protein sequence and structure. *NAR Genomics and Bioinformatics*, **6**, lqae150.

4. Alsamkary,H., Elshaffei,M., Elkerdawy,M. and Elnaggar,A. (2025) Ankh3: Multi-Task Pretraining with Sequence Denoising and Completion Enhances Protein Representations. 10.48550/arXiv.2505.20052.

5. Hayes,T., Rao,R., Akin,H., Sofroniew,N.J., Oktay,D., Lin,Z., Verkuil,R., Tran,V.Q., Deaton,J., Wiggert,M., *et al.* (2025) Simulating 500 million years of evolution with a language model. *Science*, **387**, 850–858.

6. The UniProt Consortium (2023) UniProt: the Universal Protein Knowledgebase in 2023. *Nucleic Acids Research*, **51**, D523–D531.

7. Steinegger,M. and Söding,J. (2017) MMseqs2 enables sensitive protein sequence searching for the analysis of massive data sets. *Nat Biotechnol*, **35**, 1026–1028.

8. Daily,J. (2016) Parasail: SIMD C library for global, semi-global, and local pairwise sequence alignments. *BMC Bioinformatics*, **17**, 81.

9. Alexa,A. and Rahnenfuhrer,J. (2023) topGO: Enrichment Analysis for Gene Ontology. 10.18129/B9.bioc.topGO.

10. Littmann,M., Heinzinger,M., Dallago,C., Olenyi,T. and Rost,B. (2021) Embeddings from deep learning transfer GO annotations beyond homology. *Sci Rep*, **11**, 1160.

11. Kaluza, M. C. D. P., Ramola, R., Joshi, P., Piovesan, D., Reade, W., Orchard, S., Martin, M. J., Ignatchenko, A., Participants, K. C., Rost, B., Orengo, C. A., Robinson-Rechavi, M., Durand, D., Brenner, S. E., Greene, C. S., Mooney, S. D., Friedberg, I., & Radivojac, P. (2026). Advances in Protein Function Prediction from the Fifth CAFA Challenge (p. 2026.04.27.716980). bioRxiv. <https://doi.org/10.64898/2026.04.27.716980>

12. Piovesan, D., Zago, D., Joshi,P., De Paolis, CP., Mehdiabadi,M., Ramola, R., Miguel Monzon,A., Reade,W., Friedberg, I., Radivojac, P., Tosatto, S. (2024) CAFA-evaluator: a Python tool for benchmarking ontological classification methods, *Bioinformatics Advances*, **4,** vbae043
