# Supplementary material for: FANTASIA suite: a reproducible and configurable framework for embedding-based functional annotation of proteins
Source: NAR Genom Bioinform. 2026 Sep 11;8(3):lqag106. doi: 10.1093/nargab/lqag106 (PMC13563086; doi:10.1093/nargab/lqag106)
Supplement: Supplementary file 1 [file lqag106_supplemental_files.zip › Supplementary_File_2_correctedAGO2026.docx]

**SUPPLEMENTARY MATERIAL**

**FANTASIA suite: a reproducible and configurable framework for embedding-based functional annotation of proteins**

Francisco M. Pérez-Canales^1†^, Àlex Domínguez-Rodríguez^1†,^, Belén Carbonetto^2^, Rosa Fernández^2,^*, Ildefonso Cases^1^*, and Ana M Rojas^1,^*

Francisco M. Pérez-Canales^1†^, Àlex Domínguez-Rodríguez^1†,^, Belén Carbonetto^2^, Rosa Fernández^2,^*, Ildefonso Cases^1^*, and Ana M Rojas^1,^*

^1^ Evolutionary Dynamics Department, Andalusian Center for Developmental Biology (CSIC-UPO), Seville, 41089, Spain

^2^ Metazoa Phylogenomics & Genome Evolution Lab, Institute of Evolutionary Biology (CSIC-UPF), Barcelona, 08003, Spain

* To whom correspondence should be addressed. Email: a.rojas.m@csic.es. Correspondence may also be addressed to Rosa Fernández and Ildefonso Cases.

^†^ equal contribution.

**Practical interpretation of FANTASIA predictions**

**METHODS**

**1- Data collection and construction of the evaluation panels**

Protein sequences, accessions, and taxonomic identifiers were obtained from a frozen export of the BioData PostgreSQL database used by FANTASIA. The export was generated on 27 July 2026 and retained the primary accession of each active protein with a non-null sequence and taxonomy identifier. Functional annotations and protein embeddings were taken from FANTASIA v4.1.1. GO annotations were interpreted using the GO release of 15 June 2026, and taxonomy was mapped against a frozen NCBI taxonomy snapshot.

Proteins were eligible if they had a finite embedding for all five evaluated models, a resolved accession and amino-acid sequence, and a current NCBI taxonomy identifier.

**Taxonomy identifiers.** Historical identifiers were converted to current identifiers using merged.dmp. Mouse proteins and the CAFA5 training set were excluded because they were used in separate validation analyses. Among 124,226 proteins with complete embeddings, 14,158 belonged to the mouse or CAFA5 evaluation populations and four had unresolved taxonomy, leaving 110,064 eligible proteins. Proteins with a resolved taxon but no named ancestor at a particular rank were retained in an unclassified stratum for that rank.

**Redundancy reduction.** To reduce redundancy within the evaluation set and prevent closely related proteins from being distributed across panels, sequences were clustered with MMseqs2 using a minimum sequence identity of 40% and at least 80% coverage of both sequences (--min-seq-id 0.4 -c 0.8 --cov-mode 0 --cluster-mode 1 --single-step-clustering 1). This procedure produced 54,292 connected homology clusters. One protein was selected uniformly at random from each component before stratification. The probability of selecting a particular representative was therefore $1/n_{c}$, where $n_{c}$ is the number of eligible proteins in component $c$.

**Panel construction.** Five mutually exclusive panels of 2,500 proteins were constructed from the component representatives. Genus-level blocking restricted all representatives assigned to the same genus to one panel; when a formal genus assignment was unavailable, the resolved current taxon was used as the blocking unit. Complete taxonomic blocks were assigned using a seeded balancing procedure that minimised differences in domain, phylum, annotation richness, GO-aspect composition, and sequence length. Annotation richness was grouped as 1-2, 3-5, 6-10, 11-20, or at least 21 distinct annotations, and sequence length was divided into quintiles after log transformation. GO-aspect composition represented the combination of BP, MF, and CC annotations available for each protein.

Sampling within each panel used square-root allocation, first across phyla, then across genera, and finally across the joint annotation-richness, GO-aspect, and length stratum. Compared with proportional sampling, square-root allocation reduced domination by the largest taxonomic groups while still allocating more proteins to common groups than to rare groups. Proteins were sampled randomly without replacement within the final strata, with a maximum of 256 queries per genus. The conditional inclusion probability was recorded as the fraction of available component representatives selected from each final stratum.

The resulting dataset contained 12,500 unique proteins from 12,500 distinct homology components and 1,189 genus or taxon blocks. Each panel contained 2,500 proteins and 237-239 taxonomic blocks. Panel independence was confirmed by verifying unique accessions, unique sequences, unique components, and confinement of every genus block to one panel. An all-versus-all MMseqs2 search among the selected proteins found no off-diagonal matches satisfying the 40%-identity and 80%-coverage threshold.

**2- Annotation transfer and term-level evidence**

Annotation transfer was performed separately for each embedding model and neighbourhood size. Candidate donors were first filtered to enforce reference separation. Any selected evaluation accession, any exact sequence duplicate of the query, and any donor with at least 90% global sequence identity to the query and at least 80% global alignment coverage of both the query and donor sequence was removed.

For each query, the first *k* donors in the filtered embedding-distance ranking were selected, using *k* = 1, 3, 5, 10 and 20, where required. The GO annotations associated with these donors were then pooled. A GO term was predicted for a query if it was present in at least one of its selected donors.

GO identifiers were standardized against the reference GO release before evaluation. Alternate identifiers were mapped to their primary identifiers, and obsolete terms were mapped to their designated replacements when one was available. Terms that could not be resolved and the three ontology root terms were discarded.

Annotations were expanded to include each annotation together with all of its “is_a” and “part_of” ancestors. Duplicate terms produced by multiple donors, multiple annotation records, or multiple propagation paths were collapsed.

The resulting prediction table contained one row for each distinct query-model-*k*-GO-term combination. When several of the top-*k* donors supported the same term, the donor with the smallest embedding distance was designated as the representative supporting donor. Embedding distance, global and local sequence identity, alignment coverage, and taxonomic distance were all retained from this same donor.

Evidence from the other supporting donors was retained through distinct support counts. For each predicted term, we recorded the number of supporting donor proteins and the numbers of distinct donor species, genera, and phyla.

Repeated annotation rows from the same donor did not increase these counts. Finally, a transferred term was marked as confirmed when the same resolved GO term was present in the query protein's reference annotation set. The same direct or propagated representation was applied to predictions and gold annotations before this comparison.

**3. Evidence-variable definitions**

Evidence variables were defined at the level of a predicted GO term. When a term was supported by more than one donor, all donor-specific variables below were taken from the nearest supporting donor in embedding space, as described above.

- **Embedding distance** was defined as

$$d_{\mathrm{emb}}=1-\cos\left( \mathbf{z}_{q},\mathbf{z}_{r} \right)$$

Where **z**_q_ and **z**_r_ are the query and donor embedding vectors. Smaller values therefore indicate greater embedding similarity. Embedding-distance curves used cumulative inclusion thresholds of 0.01, 0.02, 0.03, 0.05, 0.10, 0.15, 0.20, and 0.30: a point at threshold (d) retained all predicted terms with $d_{\mathrm{emb}}\leq d$.

- **Global and local sequence identity** were taken from the corresponding FANTASIA alignment fields and converted from percentages to fractions when necessary. Global query and donor coverage were calculated as the global alignment length divided by the query and donor sequence lengths, respectively. Local query and donor coverage were calculated analogously using the local alignment length.

Identity figures used the following disjoint intervals: $<20\%,$20-40%, 40-60%, 60-80%, 80-90%, and $\geq90\%$. The lower bound was included and the upper bound excluded in each intermediate interval.

- **Taxonomic distance** was derived from the current NCBI lineages of the query and representative donor. Each pair was assigned to the most specific shared rank: same species, genus, family, order, class, phylum, kingdom, or domain; pairs without a shared domain were assigned to the different-domain category. These were exact, mutually exclusive categories rather than cumulative taxonomic thresholds.
- **Donor support** was the number of distinct top-(*k*) donor proteins carrying the predicted term. Support was grouped into 1, 2, 3-4, 5-9, and at least 10 donors. The related species-, genus-, and phylum-support variables counted distinct lineages rather than donor records. Consequently, repeated annotations from one donor or multiple donors from the same lineage did not inflate the corresponding support count.

**4. Calculation of precision, recall, and F1**

Metrics were calculated separately for BP, MF, and CC, and separately for the direct and propagated GO representations. The manuscript figures use the propagated representation. A protein was evaluable for an aspect only when its reference gold annotation set contained at least one term in that aspect.

For an evaluable query protein ($q$), let ($P_{q}$) be the set of distinct GO terms predicted by a given model and value of (k), and let ($G_{q}$) be the corresponding reference gold set.

The query-level counts were:

$$TP_{q}=\left| P_{q}\cap G_{q} \right|,\quad\quad N_{q}^{\mathrm{pred}}=\left| P_{q} \right|,\quad\quad N_{q}^{\mathrm{known}}=\left| G_{q} \right|.$$

- **Query-level precision and recall** were then defined as:

$$p_{q}=\frac{TP_{q}}{N_{q}^{\mathrm{pred}}},\quad\quad r_{q}=\frac{TP_{q}}{N_{q}^{\mathrm{known}}}.$$

Precision was defined only for proteins with at least one prediction. Proteins without predictions were therefore omitted from the macro-precision denominator, but remained in the macro-recall calculation with ($r_{q}=0$). Predicted terms absent from the available gold set contributed to the precision denominator but not to ($TP_{q}$). Because GO annotation is incomplete, these terms were described as *unconfirmed* rather than as biologically false predictions.

The principal results used protein-macro averaging.

- **Macro precision and macro recall** were calculated as:

$$P_{\mathrm{macro}}=\frac{\sum_{q:N_{q}^{\mathrm{pred}}>0} p_{q}}{\left| \left\{ q:N_{q}^{\mathrm{pred}}>0 \right\} \right|}$$

$$R_{\mathrm{macro}}=\frac{\sum_{q} r_{q}}{N}$$

Where $N=\left| \left\{ q \right\} \right|$ and	$\left| \left\{ q:N_{q}^{\mathrm{pred}}>0 \right\} \right|$ is the number of proteins with at least one predicted annotation.

- **Macro F1** was calculated from the pooled macro precision and macro recall, rather than by averaging query-level F1 values:

$$F1_{\mathrm{macro}}=\frac{2P_{\mathrm{macro}}R_{\mathrm{macro}}}{P_{\mathrm{macro}}+R_{\mathrm{macro}}}$$

In the precision-recall figure 2, the highlighted point is the value of (*k*) with the largest among (*k* = 1,3,5,10,) and (20) for each model and GO aspect. $\left( F1_{\mathrm{macro}} \right)$

**5. Panel aggregation and uncertainty**

Metric numerators and denominators were first calculated separately within each of the five design panels. The pooled point estimate was then obtained by summing the panel-specific numerators and denominators before division. For example, pooled macro precision was the sum of the panel-level weighted query-precision numerators divided by the sum of their corresponding weights. This is equivalent to pooling the evaluable proteins while preserving the protein-macro definition.

Uncertainty in the plotted evidence-variable summaries was estimated by resampling whole panels with replacement.

With five panels, all $5^{5}=3,125$ ordered bootstrap resamples were enumerated. For every resample, linked metric numerators and denominators were summed across the selected panels and the ratio was recalculated. The 2.5th and 97.5th percentiles of these resampled estimates formed the 95% interval. No five-observation (t)-test was performed.

**6. Reproducibility**

All stochastic stages used the prespecified base seed 20260726. The component representative draw used the base seed, genus-to-panel assignment used a recorded derived seed, and within-panel sampling used seeds offset by 1,000 for successive panels.

The whole-panel intervals described above were deterministic because all possible ordered panel resamples were enumerated.

The analysis used FANTASIA 4.1.1, MMseqs2 18-8cc5c+ds-1, Python 3.14.4, NumPy 2.5.1, pandas 3.0.5, SciPy 1.18.0, DuckDB 1.5.5, scikit-learn 1.9.0, PyArrow 25.0.0, and Matplotlib 3.11.1. Source-file hashes, generated-data checksums, and the database and ontology snapshot identifiers were retained with the corresponding workflow outputs.

**RESULTS**

FANTASIA reports outcomes associated with annotation transfer rather than a calibrated probability that an individual prediction is correct. To provide practical guidance for interpreting these outcomes, we evaluated annotation transfer across 12,500 sequence-diverse proteins arranged in five genus-disjoint design panels. Unless otherwise indicated, the analyses below use propagated Gene Ontology (GO) annotations and protein-macro averaging, so that each evaluable protein contributes equally to the reported precision and recall.

Across all models and GO aspects, increasing the number of neighbours produced the expected precision-recall trade-off (main-text Figure 2, Figures 3A-B). Smaller neighbourhoods yielded more conservative transfers, whereas larger neighbourhoods recovered a broader fraction of known annotations at the cost of many more unconfirmed terms. In this benchmark, *k* = 1 behaved as the most conservative setting, while *k* = 3 provided a useful intermediate operating point, particularly for MF and CC. Larger neighbourhoods were most useful for recall-oriented candidate generation rather than for high-specificity annotation transfer.

The five embedding models showed the same overall response to increasing *k*, but their profiles were not identical (Supplementary Figures SF4-SF5). No single model dominated across all GO aspects and operating points. Instead, the appropriate choice depended on the intended use and on the ontology being analysed. The differences between models were modest for some conservative settings, especially for BP and CC, but were more apparent for MF.

Predictions supported by very short embedding distances were consistently enriched for agreement with the reference annotation set, whereas more permissive distance thresholds added relatively little additional support beyond the closest neighbours.

For most model-aspect profiles, an embedding distance around 0.10 marked a useful empirical transition, although this was not a universal threshold. ProtT5 showed a broader and more gradual distance-response profile, with precision continuing to decline between thresholds of 0.10 and 0.30.

**Several evidence variables** provided additional empirical stratification of annotation agreement (main-text Figure 3). Taxonomic and sequence similarity showed related but distinct patterns.

**Taxonomic proximity** (main-text Figure 3C; Supplementary Figure SF7) was informative and improved annotation agreement particularly across distant-to-intermediate relationships, although the pattern was not strictly monotonic.

Global sequence identity (Figure 3E; Supplementary Figure SF8) showed its largest increase in discriminatory value between the lowest-identity categories and the 40–60% range, whereas local identity Figure 3F; Supplementary Figure SF9) retained an additional high-similarity signal.This may reflect cases in which short conserved regions, motifs, or domains support transferable molecular or cellular annotations even when full-length similarity is lower.

**Donor support** (Figure 3D; Supplementary Figure SF6) produced the clearest precision-coverage gradient. Terms supported by multiple donors were substantially more likely to agree with the reference annotations than terms supported by only one donor, and the strongest support categories showed the highest observed agreement. These high-support predictions were uncommon, however, so donor support is best interpreted as a prioritization variable rather than as a broad-coverage annotation rule.

More generally, these evidence criteria, same-species donors, high local sequence identity, or support from multiple donors, identified smaller subsets of predictions with higher empirical precision but substantially reduced annotation and protein coverage. Their practical value should therefore be assessed by considering the trade-off between precision and the proportion of predictions or proteins retained.

The same qualitative patterns were observed when the evidence variables were evaluated at **additional neighbourhood sizes** (Figure 3D; Supplementary Figure SF6). Increasing *k* expanded the available candidate set and changed the precision-recall balance, but the main stratification signals from embedding distance, donor support, taxonomic proximity, and sequence similarity remained consistent. Thus, these variables provide useful context across operating points, while the appropriate threshold depends on the selected model, GO aspect, and intended balance between precision and recall.

Taken together, these results support a tiered interpretation of FANTASIA output.

**For conservative transfer**, users should begin with *k* = 1 and prioritize predictions supported by short embedding distances, higher sequence similarity, closer taxonomic donors, or repeated donor support when *k* >1. The *k* =3 setting provides a practical intermediate solution, especially for MF and CC, **whereas larger neighbourhoods** are better suited to exploratory or **recall-oriented use**.

For instance, for downstream analyses that depend on aggregate functional signal, such as GO term enrichment of differentially expressed genes, a moderately recall-oriented regime may be preferable to the most conservative per-term setting. In such cases, *k* = 3 or *k* = 5 can increase annotation coverage while retaining empirical stratification through embedding distance, donor support, sequence similarity, or taxonomic proximity. These settings should be interpreted as enrichment-oriented annotation sets rather than as high-confidence assignments for every individual protein.

In all cases, the associated evidence variables are best used to rank or stratify predictions, not to assign calibrated probabilities of correctness. Predicted terms absent from the reference annotation set should therefore be described as unconfirmed rather than as demonstrably incorrect. The companion data use to generate the figures are in Supplementary Table ST9.

 **Supplementary Figure SF4. Macro precision by embedding-distance at different k**. Curves are cumulative: each point retains predictions with embedding distance ≤ d, and the dashed reference line marks d = 0.10. From d = 0.10 to d = 0.30, macro precision changes by no more than three percentage points in 12 of 15 model-aspect combinations; the three exceptions are Prot-T5. Whiskers show 95% whole-panel intervals using up to five independent panels.

 **Supplementary Figure SF5. Macro recall across cumulative embedding-distance thresholds at different k**. Each point retains predictions with embedding distance ≤d\leq d≤d, and the dashed reference line marks *d* =0.10. Whiskers show 95% whole-panel intervals across the five genus-disjoint design panels.

**Supplementary figure SF6. Macro precision across disjoint bins of supporting donors at** **different *k****.* Bars identify models; whiskers show 95% intervals across five independent panels.

**Supplementary Figure SF7. Macro precision across taxonomy across *k*.** Taxonomic categories are exact bins rather than cumulative inclusion sets. Bars identify models; whiskers show 95% intervals across five independent panels.

**Supplementary Figure SF8. Macro precision across disjoint global sequence identity across k.** Bars identify models; whiskers show 95% intervals across five independent panels. Higher identity indicates greater sequence similarity. No global-identity observations were available in the ≥90% bin.

**Supplementary Figure SF9**. **Macro precision across disjoint local sequence identity across k.** Bars identify models; whiskers show 95% intervals across five independent panels. Higher identity indicates greater sequence similarity.
